# The Absence of STING Ameliorates Non-Alcoholic Fatty Liver Disease and Reforms Gut Bacterial Community

**DOI:** 10.3389/fimmu.2022.931176

**Published:** 2022-06-30

**Authors:** Qiang Zhang, Qiongyun Chen, Changsheng Yan, Chunyan Niu, Jingping Zhou, Jingjing Liu, Yang Song, Fei Zhou, Yanyun Fan, Jianlin Ren, Hongzhi Xu, Bangzhou Zhang

**Affiliations:** ^1^ Department of Gastroenterology, Yancheng Third People’s Hospital (The Yancheng School of Clinical Medicine of Nanjing Medical University), Yancheng, China; ^2^ Department of Gastroenterology, Zhongshan Hospital affiliated to Xiamen University, Xiamen, China; ^3^ Department of Digestive Disease, School of Medicine, Xiamen University, Xiamen, China; ^4^ Department of Gastroenterology, Nanjing Lishui People’s Hospital (Zhongda Hospital Lishui Branch, Southeast University), Nanjing, China; ^5^ Xiamen Key Laboratory of Intestinal Microbiome and Human Health, Zhongshan Hospital affiliated to Xiamen University, Xiamen, China; ^6^ Institute for Microbial Ecology, School of Medicine, Xiamen University, Xiamen, China

**Keywords:** STING, NAFLD, intestinal bacteria, lipid metabolism, metabolic pathway

## Abstract

Non-alcoholic fatty liver disease (NAFLD) is one of the primary causes of cirrhosis and a major risk factor for hepatocellular carcinoma and liver-related death. It has been correlated with changes in the gut microbiota, which promote its development by regulating insulin resistance, bile acid and choline metabolism, and inflammation. Recent studies suggested a controversial role of the stimulator of interferon genes (STING) in the development of NAFLD. Here, we showed that as an immune regulator, STING aggravates the progression of NAFLD in diet-induced mice and correlated it with the changes in hepatic lipid metabolism and gut microbiota diversity. After feeding wild-type (WT) and STING deletion mice with a normal control diet (NCD) or a high-fat diet (HFD), the STING deletion mice showed decreased lipid accumulation and liver inflammation compared with WT mice fed the same diet. In addition, STING specifically produced this hepatoprotective effect by inhibiting the activation of CD8^+^ T cells. The gut microbiota analysis revealed significant differences in intestinal bacteria between STING deletion mice and WT mice under the same diet and environmental conditions; moreover, differential bacterial genera were associated with altered metabolic phenotypes and involved in related metabolic pathways. Overall, our findings reveal the important regulatory role that STING plays in the progression of NAFLD. In addition, the change in intestinal microbiota diversity may be the contributing factor.

## Introduction

As the most common chronic liver disease worldwide, non-alcoholic fatty liver disease (NAFLD) is considered to be the hepatic manifestation of a metabolic syndrome. The increase in its incidence is accompanied by an increasing number of patients with an end-stage liver disease requiring liver transplantation. Thus, it has brought huge medical burdens and economic pressure globally (1–4). The pathogenesis of NAFLD is still unclear, and existing studies have only confirmed that its occurrence is related to factors such as genetic predisposition, intestinal microbiome dysregulation, insulin resistance, abnormal lipid metabolism, oxidative stress, and lipotoxicity (5). To cope with the clinical challenges posed by the disease heterogeneity, the International Fatty Liver Expert Group reached a consensus on replacing the term “NAFLD” with the new disease name “metabolic-associated fatty liver disease (MAFLD)” in 2020, marking an important milestone in NAFLD-related research (6).

The function of innate immunity in the occurrence and development of non-alcoholic fatty liver (NAFL) and non-alcoholic steatohepatitis (NASH) has been confirmed by several studies recently (7, 8). As a member of the innate immune system regulator, the stimulator of interferon genes (STING) is an endoplasmic reticulum protein encoded by transmembrane protein 173 (TMEM173). Moreover, studies have confirmed the essential function of STING in innate responses to cytosolic nucleic acid ligands, particularly double-stranded DNA (dsDNA) and unique bacterial nucleic acids called “cyclic dinucleotides” (9, 10). The activation of its downstream factors TANK-binding kinase 1 (TBK1) and interferon regulatory factor 3 (IRF3) is believed to play an important role in promoting inflammation (11, 12). Therefore, it is of great significance to study the role of cyclic GMP-AMP synthase (cGAS)-STING signal in the progression of NAFLD and thus provide a potential target for clinical treatment.

In addition, gut microbiota plays a critical role in the development and progression of NAFLD. The emergence of theories such as “gut–liver axis” and “brain–gut axis” has made intestinal microecology a hot spot in scientific research. Gut microbiota-derived molecules, such as lipopolysaccharides (LPS) and bacterial DNAs, and metabolites such as short-chain fatty acids (SCFAs) impact the intrahepatic immune cell profiles, as well as the expression of proinflammatory cytokines and chemokines in the fatty liver (13). Studies on the relationship between NAFLD and gut microbiota have contributed to the understanding of the pathogenesis of NAFLD. In addition, they provide a theoretical basis for the clinical search for specific intestinal microbes to diagnose and treat NAFLD (14–16).

Although studies in the last 2 years have reported that the expression of STING in monocyte-derived macrophages is positively correlated with liver inflammation and fibrosis progression, its specific mechanism of action is still unclear (17, 18). Considering the important role of gut microbiota in both preclinical NAFLD/NASH models and patients with NASH, the role of STING and gut microbiota interaction in modulating intrahepatic inflammation is still significant to explore. Starting from the perspective of intestinal microecology, we studied the function of STING in the occurrence of NAFLD and the changes in the gut microbiota to understand the alterations and relationships among genes, diseases, and intestinal microecology.

## Materials and Methods

### Mice and Groups

In our experiment, 6-week-old male STING knockout mice and C57BL/6J wild-type mice (denoted as STING^gt^ and WT, respectively) were used. STING^gt^ was purchased from the Model Animal Research Center of Nanjing University and bred in the Xiamen University Laboratory Animal Center. Briefly, STING^gt^ was obtained by gene editing of the Tmem173 gene using CRISPR/Cas9 technology, according to the structure of the Tmem173 gene, and exon3–exon8 of the Tmem173-201 transcript was selected as the knockout region (the Ensembl URL link for the knockout gene: http://asia.ensembl.org/Mus_musculus/Gene/Summary?g=ENSMUSG00000024349;r=18:35733679-35740554). WT was provided by the Xiamen University Laboratory Animal Center. STING^gt^ and WT mice were randomly divided into four groups after 7 to 10 days of normal feeding (denoted as NCD WT, HFD WT, NCD STING^gt^, and HFD STING^gt^). These mice were fed either a normal control diet (NCD) or a high-fat diet (HFD, 60 kcal% fat), and were maintained in a pathogen-free, temperature-controlled environment under a 12-h light/dark cycle at the Xiamen University Laboratory Animal Center. All animal protocols were approved by the Institutional Animal Care and Ethics Committee. The body weight was recorded every week for 12 weeks; at the end of the experiment, the mice were sacrificed and sampled, and the liver, subcutaneous fat, and visceral epididymal fat of mice were weighed during sampling.

## Intraperitoneal Glucose Tolerance Test

During the 12th week of animal procedures, an intraperitoneal glucose tolerance test (IPGTT) was performed before sampling. The night before the experiment, the mice were replaced with a gasket and fasted for 8 h. The body weight and fasting blood glucose of mice were recorded, and 15% glucose was injected intraperitoneally at a ratio of 1.5 g/kg. Glucose levels were determined at 15, 30, 60, and 120 min after injection, and the area under the curve was calculated.

## Hematoxylin and Eosin Staining

The intact liver was excised immediately after mice were euthanized by asphyxiation, fixed in 4% paraformaldehyde, and embedded in paraffin. Next, the liver tissue was cut into 4-µm-thick sections for hematoxylin and eosin (H&E) staining. We scored the degree of hepatocyte steatosis according to the pathologic scoring method for NAFLD reported by David. Specifically, five fields were randomly selected from each liver section under the microscope for scoring (magnification 200×). The average score of each sample and slide was taken, which was primarily based on the extent of steatosis. The sections were imaged using the LEICA DM2500 microscope attached with a LEICA DMC6200 camera.

### Immunohistochemical Staining

Paraffin-embedded mouse liver blocks were cut into sections of 4 µm thickness. These were stained using rabbit polyclonal antibodies against STING (19851-1-AP; Proteintech Group) to study its expression. The sections were imaged by a LEICA DM2500 microscope attached to a LEICA DMC6200 camera.

### Western Blotting

After briefly heating and centrifuging, the protein samples were separated on 12% sodium dodecyl sulfate–polyacrylamide gel electrophoresis (SDS–PAGE) gels and subsequently transferred to a polyvinylidene fluoride (PVDF) membrane. The non-specific protein antibody-binding sites were sealed using a blocking solution. Finally, the membrane was incubated with primary and secondary antibodies sequentially, and protein bands were visualized after washing the membrane.

### Real-Time PCR

The total RNA in liver tissues was extracted using the TRIzol reagent (15596026, Invitrogen), and the concentration and qualification of RNA were measured on a NanoDrop 2000 Spectrophotometer. Next, the quantified RNA was reverse transcribed using the Hifair II 1st Strand cDNA Synthesis SuperMix for qPCR (gDNA digester plus), and the subsequent cDNA samples were subjected to real‐time PCR using the Hieff qPCR SYBR Green Master Mix (Low Rox Plus) on a 7500 Fast Real-Time PCR instrument (Applied Biosystems). The reaction conditions were 95°C for 5 min, followed by 40 cycles of 95°C for 5 s, 60°C for 30 s, and 72°C for 20 s. Relative mRNA levels were calculated using the 2^−ΔΔCT^ method. The primer sequences are listed in **Supplementary Table 1**.

### Measurement of Liver Triglyceride Levels

Frozen livers were homogenized for 2 min at 3,000 rpm using a tissue homogenizer at 4°C, and 2 µl of the supernatant was absorbed to determine the protein concentration. Moreover, liver triglyceride levels were detected using a triglyceride detection kit (A110-1, Nanjing Jiancheng Bioengineering Institute).

### Measurement of Serum Parameters

Blood samples were collected from mouse retro-orbital bleeding and placed at room temperature for 1 h. After centrifugation at 3,500 rpm for 10 min, serum samples were collected for determining alanine aminotransferase (ALT) and aspartate aminotransferase (AST) levels using an alanine aminotransferase kit (105-000442-00, IFCC Method, Mindray) and an aspartate aminotransferase kit (105-000443-00, IFCC Method, Mindray), respectively, on an automatic biochemical analyzer (BS-240vet, Mindray). The levels of inflammatory factors (tumor necrosis factor [TNF]-α, interleukin [IL]-1β, and IL-6) in the serum of mice were measured using enzyme-linked immunosorbent assay (ELISA) kits (Invitrogen) in accordance with the manufacturer’s protocols.

### Flow Cytometry

Liver samples were rinsed with phosphate-buffered saline (PBS) and then treated with collagenase D (0.75 mg·ml^−1^) at 37°C for 30 min. The reaction was stopped by adding preheated ethylenediaminetetraacetic acid (EDTA) containing the flow cytometry staining (FACS) buffer (PBS, 10% fetal bovine serum [FBS], 0.1% NaO_3_, 5 mM EDTA). Cells were stained for flow cytometry using the corresponding antibodies and acquired by flow cytometry (FACS Fortessa; BD Bioscience), and data were analyzed using the FlowJo software.

### DNA Extraction and 16S rDNA Bioinformatics Analysis

Total DNA of cecum (Proximal) and distal colon (Distal) contents was extracted using the QIAamp Power Fecal DNA kit (12830, Qiagen). The extracted DNA was amplified by polymerase chain reaction (PCR) using bar-coded universal primers 515 F (F: forward primer, 5’-GTGYCAGCMGGCCGCGGTAA-3’) and 806 R (R: reverse primer, 5’-GGACTACNVGGGTWTCTAAT-3’). Next, high-throughput sequencing was conducted using standard protocols on an Illumina MiniSeq platform. Bioinformatics analysis followed the steps in our previous studies (19, 20). Briefly, the FLASH software was used to trim raw sequencing reads (21). The high-quality reads were checked for chimeras and clustered into operational taxonomic units (OTUs, 97% nucleotide sequence identity) by USESARCH (22). OTU representative sequences were finally aligned to the SILVA database to obtain bacterial taxonomic classification by the RDP Classifier (23). All samples were rarefied to 28,973 reads per sample for downstream analysis.

### Statistical Analysis

GraphPad Prism 8.0 and R 3.3.2 mainly with packages of vegan (24–27) were used for analysis and figure visualization. Data are expressed as mean ± standard error of the mean (SEM). Differences were analyzed by one-way analysis of variance (ANOVA) or permutational multivariate analysis of variance (PERMANOVA) for multiple group comparison. A *p*-value < 0.05 was considered significant.

## Results

### STING Deficiency Downregulates Body Weight, Liver Weight, and Fat Weight in NAFLD Mice

To study the function of STING in NAFLD, STING^gt^ mice were used under HFD-fed conditions. We first verified the genotype of STING^gt^ mice at the protein level. The results showed that under the same conditions as that of the internal control, the protein expression of STING in the liver of STING^gt^ mice was significantly decreased compared with that in the WT mice (**Supplementary Figure 1**).

**Figure 1:**
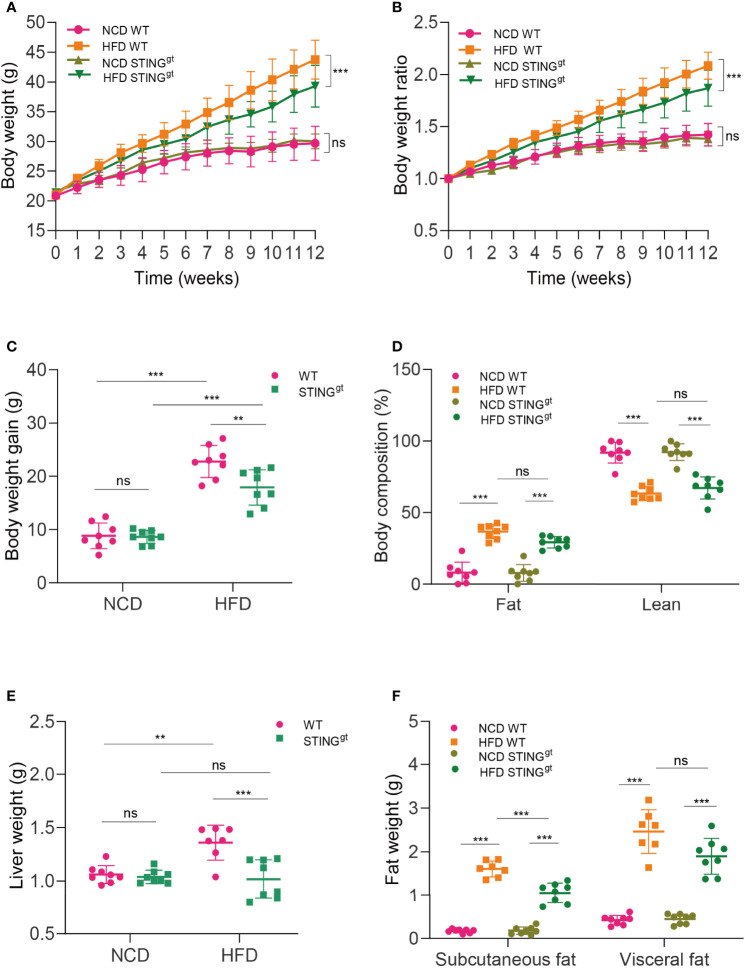
STING deficiency downregulates body weight and liver and fat weight in NAFLD mice. WT and STING^gt^ mice were fed NCD or HFD for 12 weeks at 6 weeks of age. **(A–C)** Body weight, body weight ratio, and body weight gain were determined. **(D)** Body composition (fat and lean content) was also measured. **(E, F)** Measurements of liver and fat weight (subcutaneous fat and visceral epididymal fat) at 12 weeks. ns, no significance; NCD, normal chow diet; HFD, high-fat diet; WT, wild-type; STING^gt^, STING-deletion mice. Data are expressed as mean ± SD, *n* = 6–8 per group. **p < 0.01, ***p < 0.001 (unpaired *t*-test or ANOVA).

Under NCD-fed conditions, STING^gt^ mice exhibited no significant difference from WT mice in most parameters related to NAFLD. Upon HFD feeding for 12 weeks, mice revealed overt NAFLD aspects. In particular, STING^gt^ mice displayed a smaller gain in body weight compared with WT mice (**Figures 1A–C**). Moreover, body composition was analyzed using EchoMRI after the feeding period. The body composition analysis indicated higher fat mass and lower muscle mass in HFD-fed mice; however, the values of these in HFD-fed STING^gt^ mice did not differ significantly from those in HFD-fed WT mice (**Figure 1D**).

To examine the NAFLD aspects, we weighed the liver, subcutaneous fat, and visceral epididymal fat of mice. Statistically, the liver weight of HFD-fed WT mice was higher than that of HFD-fed STING^gt^ mice. Consistently, HFD-fed STING^gt^ mice displayed a significant decrease in the weight of adipose tissue compared with HFD-fed WT mice (**Figures 1E, F**).

With regard to metabolic homeostasis, the intraperitoneal glucose tolerance test (IPGTT) in HFD-fed mice revealed a higher glucose tolerance than that in NCD mice over time. Interestingly, we found no significant difference in blood glucose levels between HFD-fed WT and HFD-fed STING^gt^ mice (**Supplementary Figures 2**, **3**). These results suggested that STING plays a pernicious role in NAFLD.

### STING Deficiency Alleviate HFD-Induced Steatohepatitis and Liver Injury

**Figure 2:**
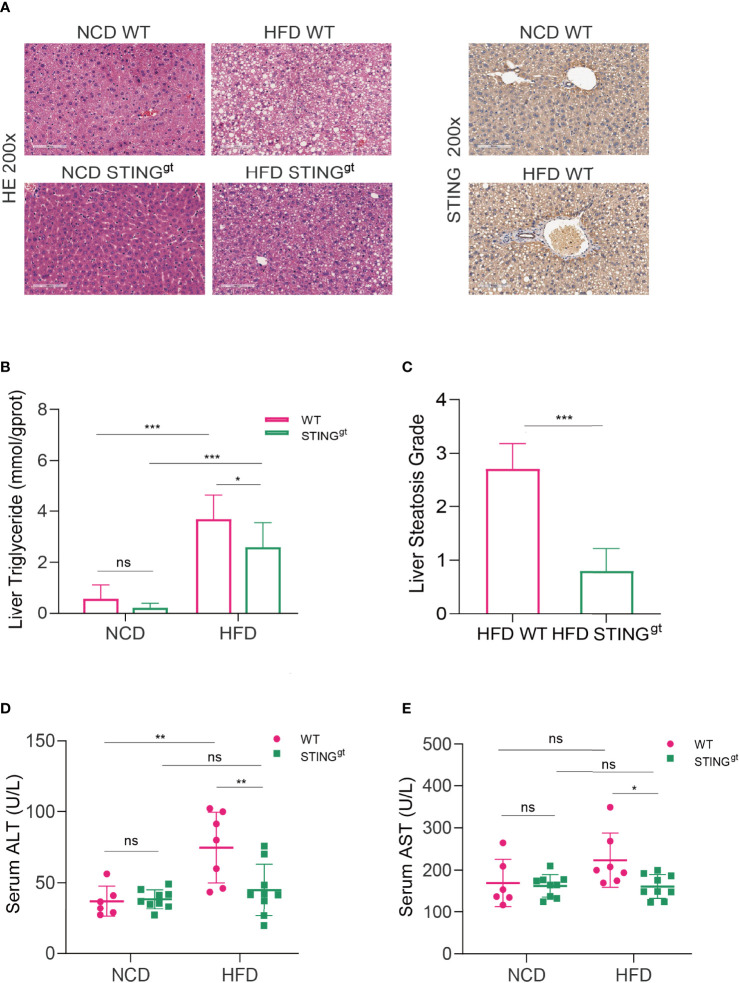
STING deficiency alleviates HFD-induced steatohepatitis and liver injury. The liver and blood of mice were collected at 12 weeks. **(A)** Representative images of liver sections stained with HE and immunohistochemistry of STING. **(B, C)** Liver triglyceride content and hepatic steatosis scores were determined. **(D, E)** Serum levels of ALT and AST were measured. ns, no significance; ALT, alanine aminotransferase; AST, aspartate aminotransferase. Data are expressed as mean ± SD, *n* = 6–8 per group. *p < 0.05, **p < 0.01, ***p < 0.001 (unpaired *t*-test or ANOVA).

Pathological staining of liver tissue sections is the gold standard for the diagnosis and efficacy evaluation of NAFLD. We histologically confirmed hepatic steatosis in each group of mice by H&E staining. Significant steatohepatitis characterized by steatosis and lobular inflammation was observed in WT mice fed with HFD for 12 weeks, whereas only minor steatosis was found in STING^gt^ mice fed with the same diet (**Figure 2A**). In agreement with these results, the concentrations of liver triglycerides and NAFLD activity score (NAS) testified that the degree of liver steatosis in STING^gt^ mice was significantly lower than that in WT mice fed with HFD (**Figures 2B, C**). In addition, the expression of STING in the liver of the HFD group was significantly increased compared with that in the NCD group in WT mice. Based on the previous results, we inferred that STING is involved in the occurrence of NAFLD (**Figure 2A**).

To further prove the function of STING in the inflammatory reaction of NAFLD, the development of hepatic injury in individual groups was confirmed, as indicated by significantly decreased ALT and AST levels compared with those of the pair-fed group (**Figures 2D, E**). We further assessed the mRNA levels of inflammatory factors in the liver of the HFD group; the results showed that mRNA levels of inflammatory cytokines, such as TNF-α, IL-1α, IL-1β, and interferon (IFN)-γ, were significantly decreased in the STING^gt^ mice compared to the HFD-fed WT mice (**Supplementary Figure 4**). Altogether, our findings indicated the ameliorative effects of STING deletion against the development of NAFLD by suppressing both hepatic steatosis and inflammation.

### STING Deficiency Inhibit Hepatic and Systemic Inflammatory Activation After HFD Challenge

**Figure 3:**
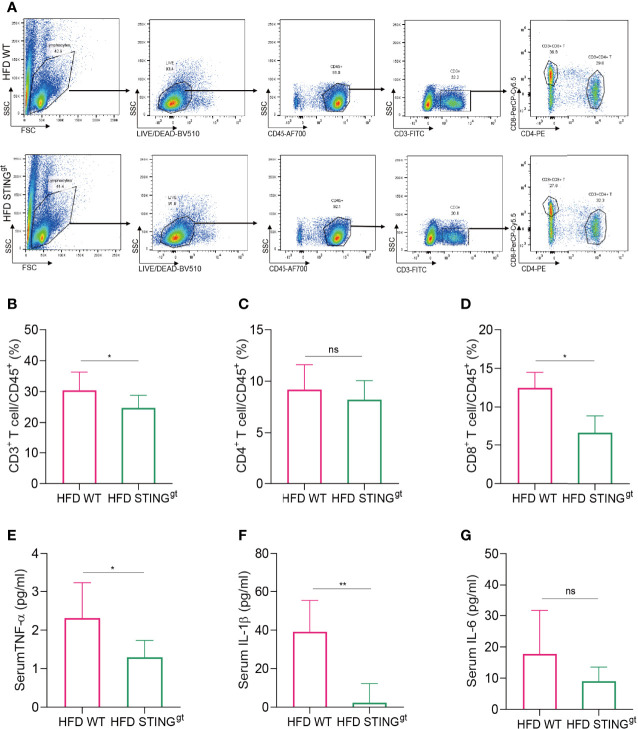
STING deficiency inhibits hepatic and systemic inflammatory activation after HFD challenge. The liver and blood of mice were collected at 12 weeks. **(A)** Flow cytometry analysis of the representative histogram and antibody labeling. **(B–D)** The proportion of CD3^+^ T cells, CD4^+^ T cells, and CD8^+^ T cells in CD45^+^ T cells, respectively. **(E–G)** Serum levels of TNF-α, IL-1β, and IL-6 were determined. ns, no significance. Data are expressed as mean ± SD, *n* = 6–8 per group. *p < 0.05, **p < 0.01 (unpaired *t*-test or ANOVA).

Liver-produced IFN-I is known to promote the occurrence and development of HFD-induced NAFLD in mice, which is specifically related to the activation of CD8^+^T cells and leads to insulin resistance and liver gluconeogenesis (28). Therefore, we investigated the changes in liver T lymphocytes and the proportion of CD8^+^T cells in HFD-fed mice (**Supplementary Table 2**). According to the results, the levels of CD3^+^T cells in STING^gt^ mice were significantly lower than those in WT mice that fed on HFD. No significant difference was noted in the number of CD4^+^T cells between the two groups, whereas the changing trend in the number of CD8^+^T cells between the two groups was consistent with that of CD3^+^T cells with a statistical difference (**Figures 3A–D**). The above results indicated that the liver immune response is partially suppressed in the absence of STING, primarily by inhibiting the activation of CD8^+^T cells to produce this hepatoprotective effect.

Next, we detected the levels of serum inflammatory cytokines in HFD-fed mice and found that serum TNF-α and IL-1β levels in the STING^gt^ group were significantly lower than those in the WT group. No significant difference was observed in the levels of IL-6 between the two groups; however, the levels in the STING^gt^ group were lower than those in the WT group (**Figures 3E–G**), suggesting that STING deletion inhibited the inflammatory damage in HFD-induced NAFLD.

### STING Deficiency Affects the Biodiversity of Intestinal Microbiota in Mice

**Figure 4:**
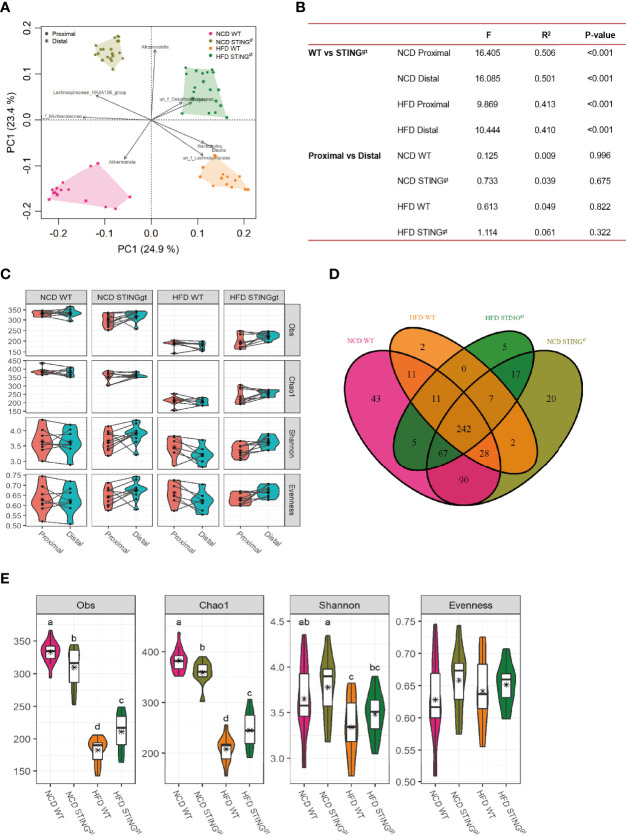
The effect of STING on biodiversity of gut microbiota. **(A)** OTU Venn analysis of samples in different groups. **(B)** The principal coordinate analysis (PCoA) of all samples at the OTU level; the points represent samples, and shapes represent the different group. **(C)** The changes of α-diversity (including Obs, Chao1, Shannon, and Evenness) in each group. **(D)** Comparison of microbiota α-diversity between the proximal and distal colons of mice. **(E)** Statistical analysis of differences in gut microbiota among different diets, genotypes, and colonic segments, with letters indicating the grouping and stars representing the mean values. Obs, observed species.

Microbial composition and diversity were analyzed by 16S rRNA gene sequencing for samples from the cecum and distal colon. The principal component analysis (PCA) revealed distinct clustering of microbial composition among the four groups, and significant differences among wild-type and the STING^gt^ mice (**Figure 4A**), while no significant differences were observed between proximal and distal intestinal content. We further analyzed the microbial diversity from proximal and distal sources separately. No significant difference was observed in the biodiversity between the two sites in the same group. Interestingly, there were significant differences in the gut microbiota at the same site between WT and STING deletion mice under the same diet (**Figures 4B, C**), which partly indicated that the diversity of the intestinal microbial community was related to the diet and genotype, but not to the location of the colon.

Finally, combining the two-site microbiota. Venn diagrams depicted considerable intestinal microbiota diversities in the NCD group compared with the HFD group. The composition of the bacterial community could be partially changed by diet. A total of 550 OTUs were obtained in the intestinal bacterial community in the four groups, among which 242 OTUs were shared by the four groups (**Figure 4D**). Compared with the NCD, the abundance of microbial components and Shannon’s index decreased significantly after HFD feeding. Interestingly, the richness, evenness, and Shannon’s index of intestinal microbiota in the HFD-fed STING^gt^ mice were higher than those in the WT mice under the same diet (**Figure 4E**), which was consistent with the severity of NAFLD in mice, suggesting that the diversity of intestinal microbiota is related to the improvement in NAFLD.

### Prediction and Analysis of Intestinal Microbiota Composition and Metabolic Pathway Function in Mice

**Figure 5:**
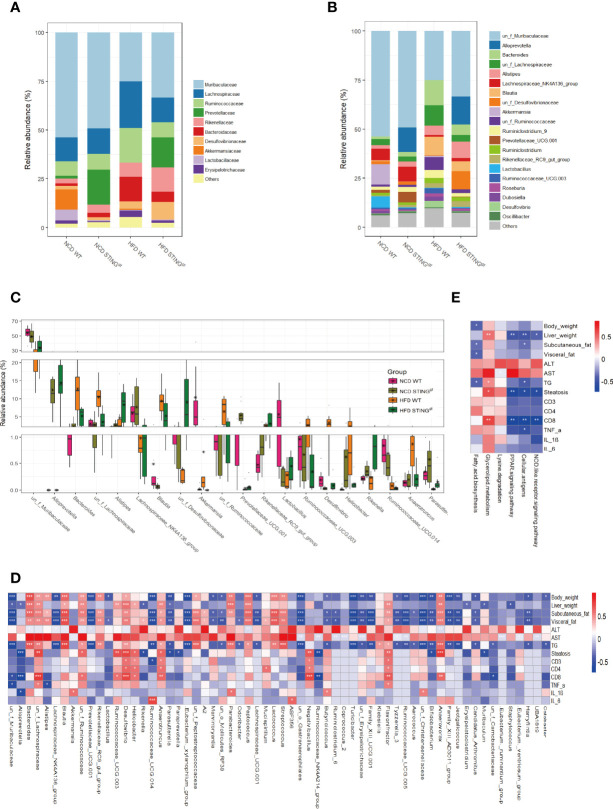
STING deficiency leads to the alteration of gut microbiota components and metabolic phenotype-related pathway in mice. **(A, B)** Bar charts represent the relative abundance of bacterial families and genus in all mice according to their diet and genotype. **(C)** The relative abundance of different species in the gut microbiota at the genus level of WT and STING^gt^ mice under different diets (Top 20). **(D)** Correlation analysis of the phenotype of NAFLD and gut microbiota at the genus level. **(E)** Correlation analysis of differential metabolic pathways associated with NAFLD development and metabolic phenotype. *p < 0.05, **p < 0.01, ***p < 0.001 (unpaired t-test or ANOVA).

Based on the above analysis results, we further studied the distribution of the relative abundance of intestinal microbiota in mice at different taxonomic levels. We found differences at the class level (**Supplementary Figure 5**), which are consistent with those mentioned in the literature (15, 29, 30). At the family level, the abundances of *Muribaculaceae*, *Akkermansiaceae*, *Prevotellaceae*, and *Lactobacillaceae* in WT mice decreased significantly, whereas the relative abundances of *Lachnospiraceae*, *Rikenellaceae*, and *Bacteroidaceae* increased significantly, among which *Akkermansiaceae* and *Lactobacillaceae* were typical representatives of probiotics; the decline in their relative abundance is mostly related to disease status. Furthermore, we analyzed the effect of genotype on intestinal microecology and found that the relative abundance of *Prevotellaceae* in STING^gt^ mice was distinctly higher than that of WT mice on the same diet. However, the relative abundances of *Akkermansiaceae* and *Lactobacillaceae* in the intestine of STING^gt^ mice were extremely low, regardless of the diet, which may be attributed to the decomposition into bacterial products while exerting their metabolic effects and therefore cannot be detected (**Figure 5A**). Similarly, we analyzed the composition of the microbiota at the genus level. Although the two different genotypes of mice had the same tendency to alter the composition of intestinal microbiota caused by dietary changes, they still showed their characteristics of changes in the intestinal flora. Interestingly, we found that compared with WT mice on the same diet, the relatively beneficial bacteria in the gut microbiota of STING-deficient mice were more predominant and thus protected against NAFLD. Furthermore, different species were ranked from high to low in terms of relative abundance, and there were significant differences in the abundance of gut microbiota among the four groups of mice (**Figures 5B, C**).

Finally, we analyzed the correlations between the phenotypes of NAFLD and gut microbiota at the genus level, and found 65 distinct bacterial genera with significantly different disease phenotypes. Further functional prediction of differential bacteria revealed that lipid metabolism and immune response-related microbiota functions were significantly correlated with disease phenotypes, and the involvement of certain other pathways was also associated with NAFLD progression, which include NOD-like receptor signaling pathway, fatty acid biosynthesis, and lysine degradation pathway (**Figures 5D, E**, **Supplementary Figures 6, 7**).

## Discussion

Recent years have witnessed an increase in the number of studies on the relationship between intestinal microecology and host health, largely focusing on the effects of intestinal microbiota or specific strains and its metabolites on human metabolism, immunity, and even tumor development (31–33). The function of intestinal microbes in the pathophysiology of NAFLD through the gut–liver axis is a hot topic in the field of metabolic research (34, 35). However, there exist a few studies related to the change in the gut microbiota caused by changes in the host genotype and disease phenotype; most of them are correlation studies. We primarily explored whether there exists a potential connection between the cGAS–STING signaling pathway and the occurrence of NAFLD and the changes in the intestinal bacteria during this process. Furthermore, we attempted to identify bacteria with differences after host genetic changes and their effects on NAFLD and metabolic pathways. Based on the results obtained, we can preliminarily confirm that the absence of STING alleviates hepatic steatosis and inflammation in NAFLD induced by HFD. In addition, the appearance of this phenotype is accompanied by changes in the gut microbiota and enrichment of related metabolic pathways. We also found that differential bacterial genera were associated with altered metabolic phenotypes and were involved in related metabolic pathways.

The cGAS–STING pathway is crucial in recognizing DNA molecules derived from multiple sources and subsequently initiating the innate immune response (12, 36). Although STING has been reported to be associated with several inflammatory diseases such as ulcerative colitis (IBD) and systemic lupus erythematosus (SLE), the specific mechanism has remained elusive (37–40). Studies on the relationship between cGAS/STING/TBK1 and distant metastasis of tumors have confirmed that the activation of cGAS/STING/TBK1 inhibits tumor metastasis by synthesizing IFN and recruiting NK cells and macrophages (41, 42). A recent study published in *Cancer Cell* revealed that *p53* mutant can directly bind to the downstream molecule TBK1 of STING, thereby inhibiting the formation of the STING/TBK1/IRF3 complex and enabling the immune escape of tumor cells (43). In the study on the relationship between STING and NAFLD, our results showed that the expression of STING in the liver of NAFLD mice increased significantly, and the liver phenotype was improved when STING was absent, indicating the promotion of STING in the occurrence of NAFLD. More interestingly, while investigating how changes in gut microecology affect the changes in the genotype, we found that the intestinal bacteria also have significant differences between the STING^gt^ mice and WT mice under the same diet and environmental conditions, illustrating that host genotype is crucial to the shaping of intestinal microbiota, except for environment and diet, and this change in the gut microbiota can affect the development of NAFLD.

The variation in the intestinal microecology caused by metabolic changes has been related to the occurrence of NAFLD because NAFLD is considered to be the liver manifestation of the metabolic syndrome. It can be predicted that liver disease is related to the imbalance in the intestinal microbiota. Previous studies on the distribution characteristics of gut microbiota in NAFL or NASH patients and healthy people reported that at the family level, compared with normal healthy people, the relative abundances of *Rikenellaceae* and *Ruminoccaceae* in the intestine of patients with NAFLD increased, whereas that of *Enterobacteriaceae* decreased. At the genus level, intestinal bacteria in patients with NAFLD showed an increase in the abundance of *Escherichia* and a decrease in the abundance of *Prevotella* (44, 45), which is consistent with the changes in the intestinal microbiome found in our study. In addition, studies have revealed the mechanisms through which the disruption of the gut microbiota contributes to the development of NAFLD. In mice, an HFD induces a diet-driven dysbiosis that promotes the damage to the gut–vascular barrier and bacterial translocation to the liver, and bacteria-derived LPS induces hepatic TNF-α production, consequently stimulating hepatocyte apoptosis (46, 47). The correlation analysis, based on the role of bacteria in NAFLD phenotype, revealed significant differences in the abundance of genera among different groups, including *Butyricicoccus* and *Klebsiella*. It is positively or negatively correlated to the disease phenotype. These studies indicate that although the appearance of NAFLD is related to several factors such as environment, genetics, and metabolism, the variation in the components of intestinal flora can reveal and even affect the occurrence of NAFLD to a certain extent.

Our study has some shortcomings. First, we focused on the variations in the intestinal microbiota after the change in the genotype and their impact on the occurrence and development of NAFLD; the specific mechanism of STING promoting the evolution of NAFLD disease is still not clear. In addition, we could not identify strains that play a key role in the changes in intestinal microecology and link them with STING to elucidate the mechanism of NAFLD-related phenotypic variations, which is the main direction in which future work needs to be done.

## Data Availability Statement

The datasets presented in this study can be found in online repositories. The name of the repository and accession number can be found below: NCBI Sequence Read Archive; accession number PRJNA841779.

## Ethics Statement

The animal study was reviewed and approved by Laboratory Animal Ethics Committee Xiamen University.

## Author Contributions

QZ, QC, and CY designed the project, performed experiments, and collected and analyzed the data. QZ and CN wrote the manuscript. CN and JZ helped modify and revise the article. JL and YS worked on the mouse model. FZ and YF maintained and genotyped the mice. JR, HX, and BZ supervised and coordinated the work, and designed the overall research study. All authors contributed to the article and approved the submitted version.

## Funding

This project was supported by the National Natural Science Foundation of China (81800517), the Fujian Provincial Natural Science Foundation (2020J01122587), the Xiamen Key Programs of Medical Health (3502Z20204007), and the Xiamen Priority Programs of Medical Health (3502Z20199172).

## Conflict of Interest

The authors declare that the research was conducted in the absence of any commercial or financial relationships that could be construed as a potential conflict of interest.

## Publisher’s Note

All claims expressed in this article are solely those of the authors and do not necessarily represent those of their affiliated organizations, or those of the publisher, the editors and the reviewers. Any product that may be evaluated in this article, or claim that may be made by its manufacturer, is not guaranteed or endorsed by the publisher.
