# Supplementary material for: The Absence of STING Ameliorates Non-Alcoholic Fatty Liver Disease and Reforms Gut Bacterial Community
Source: Front Immunol. 2022 Jun 30;13:931176. doi: 10.3389/fimmu.2022.931176 (PMC9279660; doi:10.3389/fimmu.2022.931176)
Supplement: Supplementary file 1 [file DataSheet_1.pdf]

Supplementary Tables

Supplementary Table 1: The primer sequence of inflammatory factors.

Gene name	Forward primer	Reverse primer
GAPDH	CATCACTGCCACCCAGAAGACT	ATGCCAGTGAGCTTCCCGTTCAG
IL-1α	ACGGCTGAGTTTCAGTGAGACC	CACTCTGGTAGGTGTAAGGTGC
IL-1β	TGGACCTTCCAGGATGAGGAC	GTTCATCTCGGAGCCTGTAGTG
TNF-α	CCTGTAGCCCACGTCGTAG	GGGAGTAGACAAGGTACAACCC
IFN-γ	GATGCATTCATGAGTATTGCCA	GTGGACCACTCGGATGAGCTC
TGF-β	TGACGTCACTGGAGTTGTACGG	GGTTCATGTCATGGATGGTGC

Supplementary Table 2: The total cell number of each subsets in liver lymphocytes.

Group / Cell subsets	HFD WT	HFD STING^{gt}
CD45+ T cells	37057	36564
CD3+ T cells	11764	9998
CD4+ T cells	3207	2807
CD8+ T cells	4955	2636

Supplementary Figures

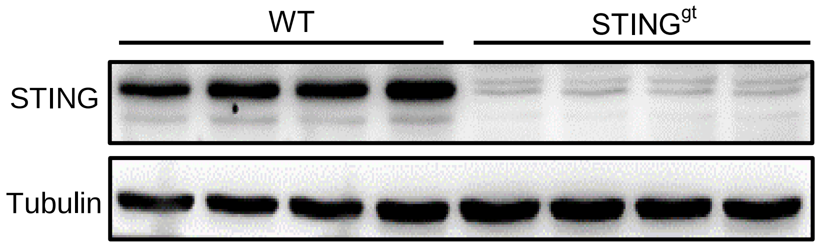

Supplementary Figure 1: Protein level of liver STING. The hepatic level of STING protein in different group mice fed with HFD for 12 weeks, four samples were randomly selected from each group. n=4 per group.

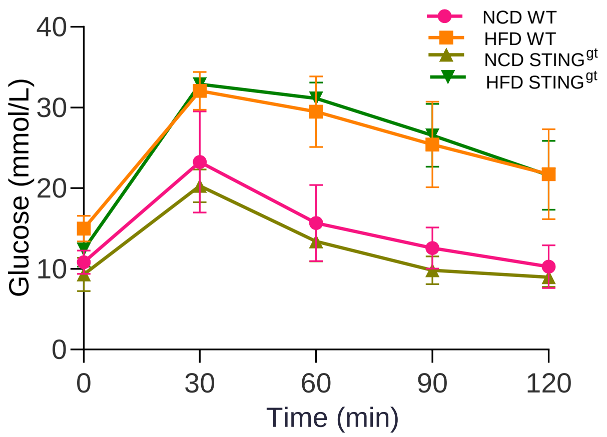

Supplementary Figure 2: Intraperitoneal Glucose Tolerance Test(IPGTT). The IPGTT blood glucose curve of mice fed with normal control diet or high fat diet for 12 weeks. n=6-8 per group.

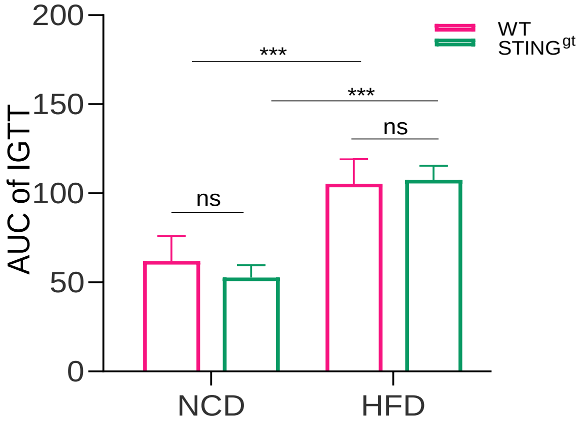

Supplementary Figure 3: The area under the curve of IPGTT. The area under the blood glucose curve in mice. Data are expressed as mean ± SD,n=6-8 per group. *p <0.05, **p <0.01, ***p <0.001 (unpaired t test or ANOVA).

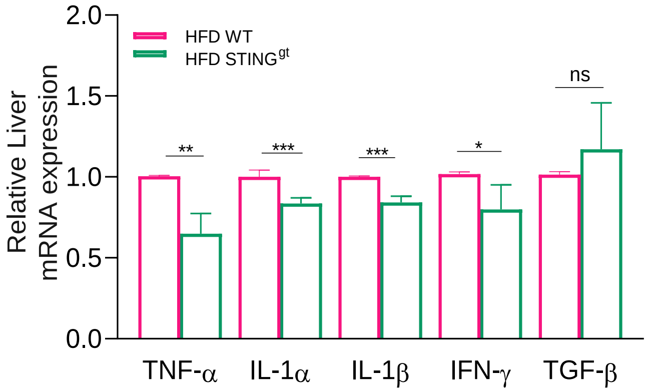

Supplementary Figure 4: The mRNA level of hepatic inflammatory factor. After 12 weeks of high-fat diet, the mRMA level of hepatic inflammatory factor were detected. Data are expressed as mean ± SD,n=6-8 per group. *p <0.05, **p <0.01, ***p <0.001 (unpaired t test or ANOVA).

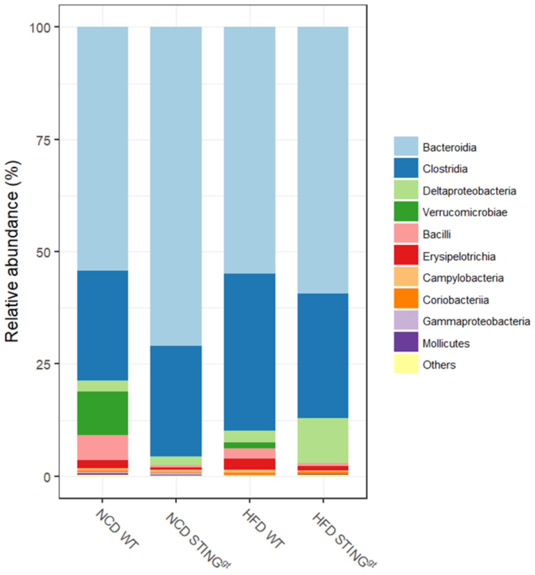

Supplementary Figure 5: Bar charts of relative abundance at class level. Bar charts of relative abundance of intestinal bacteria at class level in WT and STING^{gt} mice at 12 weeks under different diets.

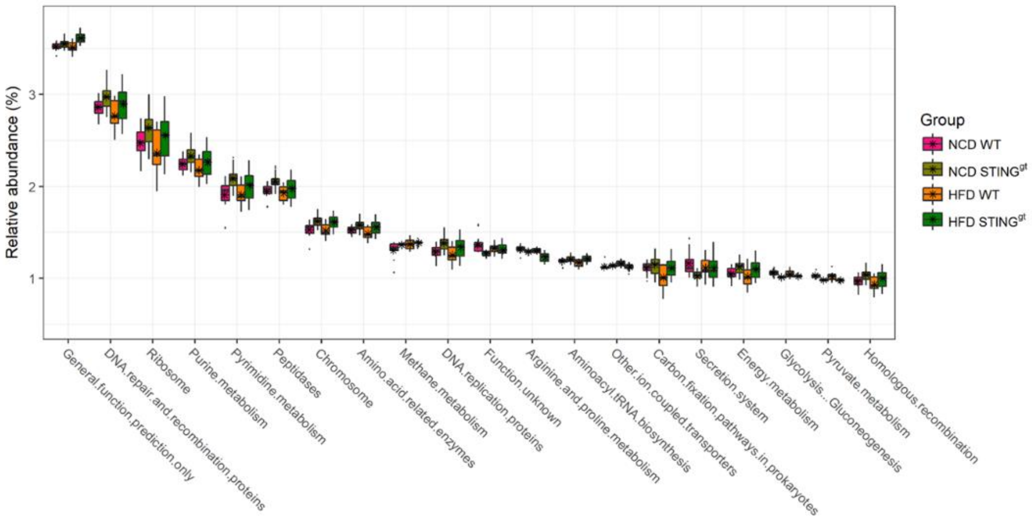

Supplementary Figure 6: Functional prediction of metabolic pathways of intestinal microbiota. Relative abundance of pathways involved in functional prediction of metabolic pathways in WT and STING^{gt} mice at 12 weeks under different diets(Top 20).

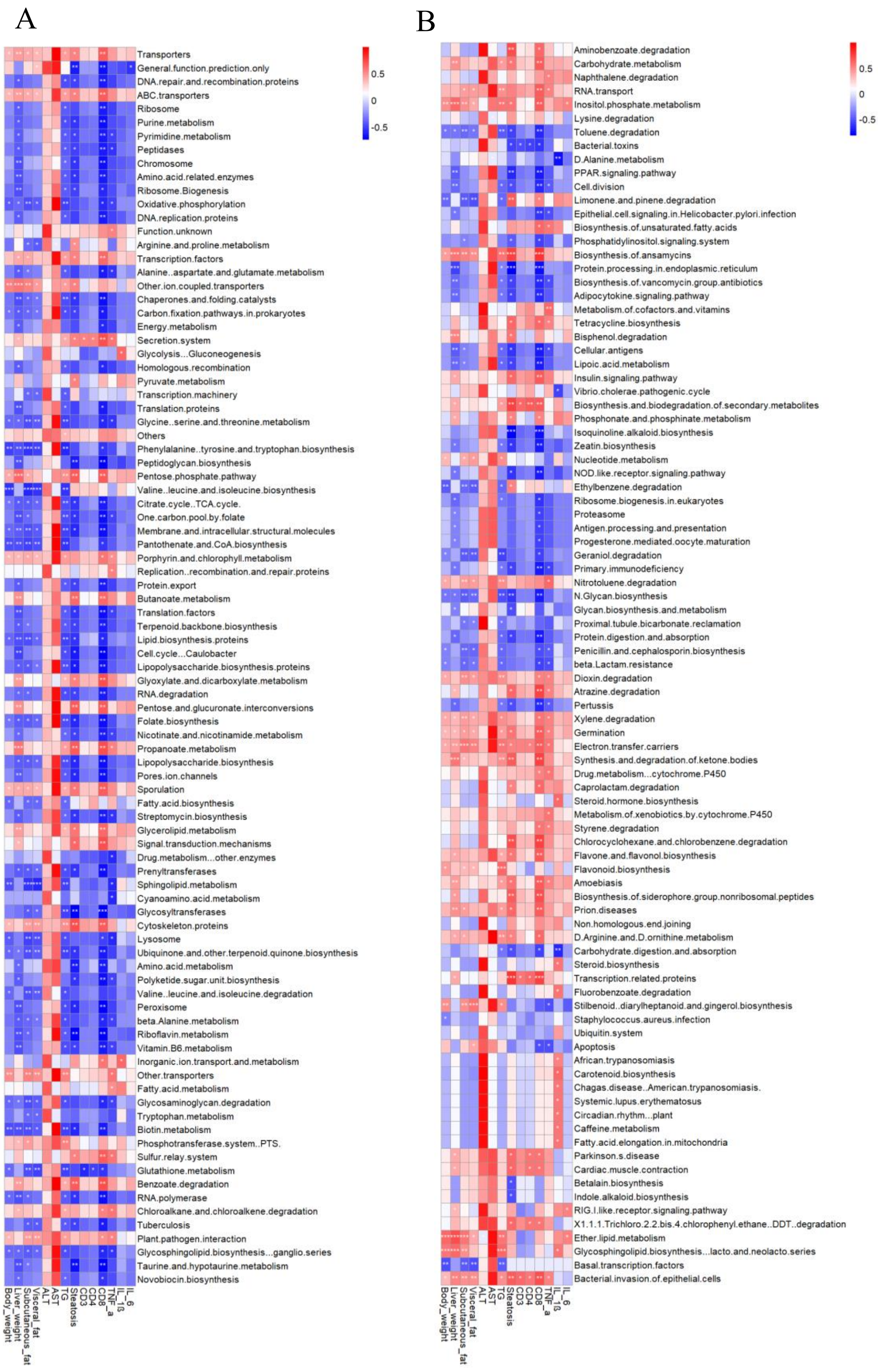

Supplementary Figure 7: Correlation analysis of differential metabolic pathways, top 1-91 (A) and top 91-182 (B) in relative abundance, associated with metabolic phenotype.
